# Supplementary material for: A yeast phenomic model for the influence of Warburg metabolism on genetic buffering of doxorubicin
Source: Cancer Metab. 2019 Oct 23;7:9. doi: 10.1186/s40170-019-0201-3 (PMC6806529; doi:10.1186/s40170-019-0201-3)
Supplement: Supplementary file 9 — Additional file 9. GO term-specific heatmaps for REMc/GTF-enriched clusters. GO term-specific heatmaps for significant GO process terms were generated as described in methods and Figs. 3 and 4. Any related child terms are presented in subsequent pages of the parent file name. GO terms with more than 100 children, with 2 or fewer genes annotated to the term, or a file size over 300KB are not shown. All heatmaps are generated with the same layout (see Figs. 3 and 4). [file 40170_2019_201_MOESM9_ESM.bz2 › Additional_File9_GOTermHeatmaps/Additional_File9_GOTermHeatmap/RNA_localization.pdf]

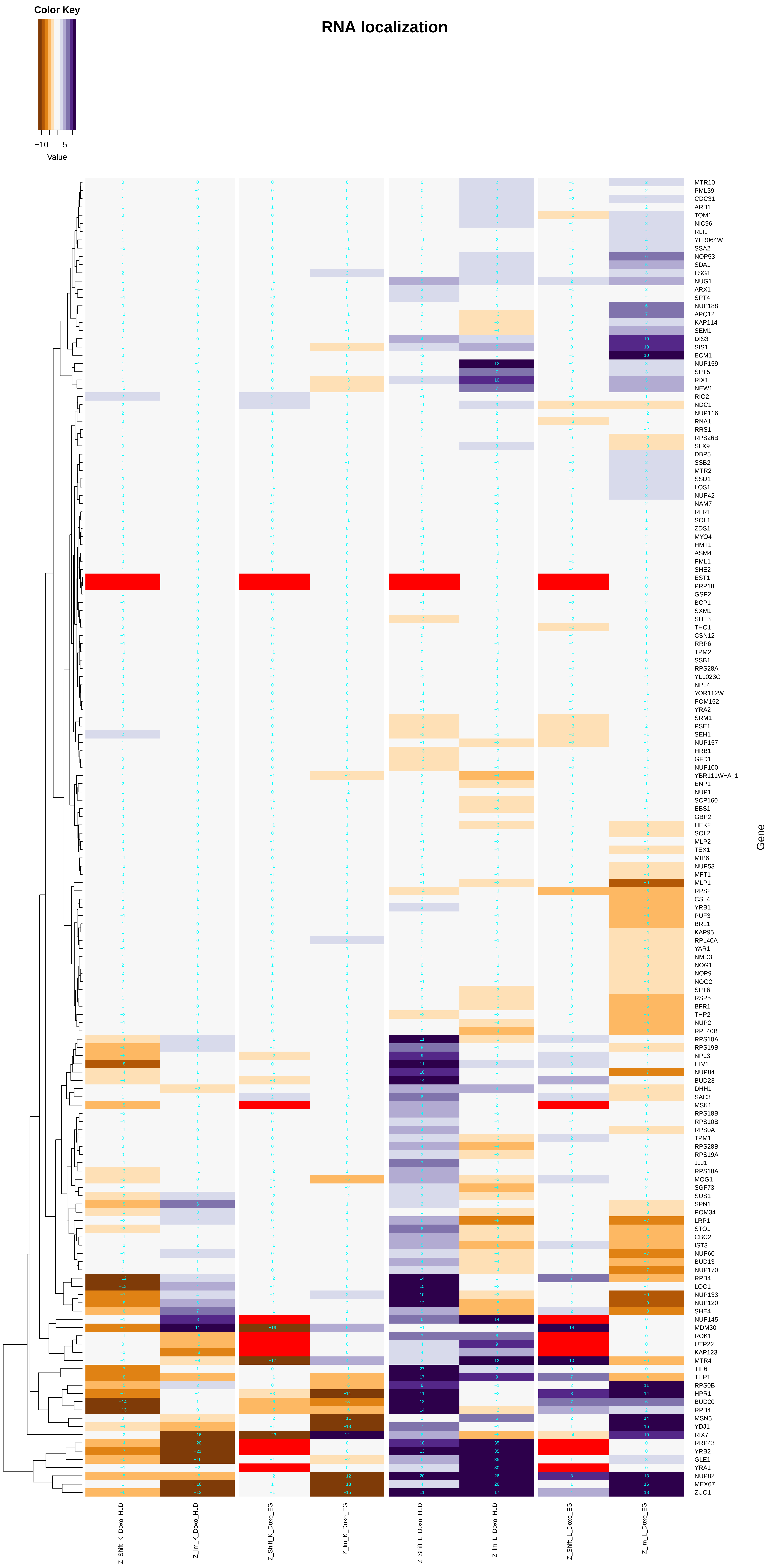

Color Key
−10
5
Value
RNA localization
MTR10
PML39
CDC31
ARB1
TOM1
NIC96
RLI1
YLR064W
SSA2
NOP53
SDA1
LSG1
NUG1
ARX1
SPT4
NUP188
APQ12
KAP114
SEM1
DIS3
SIS1
ECM1
NUP159
SPT5
RIX1
NEW1
RIO2
NDC1
NUP116
RNA1
RRS1
RPS26B
SLX9
DBP5
SSB2
MTR2
SSD1
LOS1
NUP42
NAM7
RLR1
SOL1
ZDS1
MYO4
HMT1
ASM4
PML1
SHE2
EST1
PRP18
GSP2
BCP1
SXM1
SHE3
THO1
CSN12
RRP6
TPM2
SSB1
RPS28A
YLL023C
NPL4
YOR112W
POM152
YRA2
SRM1
PSE1
SEH1
NUP157
HRB1
GFD1
NUP100
YBR111W−A_1
ENP1
NUP1
SCP160
EBS1
GBP2
HEK2
SOL2
MLP2
TEX1
MIP6
NUP53
MFT1
MLP1
RPS2
CSL4
YRB1
PUF3
BRL1
KAP95
RPL40A
YAR1
NMD3
NOG1
NOP9
NOG2
SPT6
RSP5
BFR1
THP2
NUP2
RPL40B
RPS10A
RPS19B
NPL3
LTV1
NUP84
BUD23
DHH1
SAC3
MSK1
RPS18B
RPS10B
RPS0A
TPM1
RPS28B
RPS19A
JJJ1
RPS18A
MOG1
SGF73
SUS1
SPN1
POM34
LRP1
STO1
CBC2
IST3
NUP60
BUD13
NUP170
RPB4
LOC1
NUP133
NUP120
SHE4
NUP145
MDM30
ROK1
UTP22
KAP123
MTR4
TIF6
THP1
RPS0B
HPR1
BUD20
RPB4
MSN5
YDJ1
RIX7
RRP43
YRB2
GLE1
YRA1
NUP82
MEX67
ZUO1
Gene
Z_Shift_K_Doxo_HLD
Z_lm_K_Doxo_HLD
Z_Shift_K_Doxo_EG
Z_lm_K_Doxo_EG
Z_Shift_L_Doxo_HLD
Z_lm_L_Doxo_HLD
Z_Shift_L_Doxo_EG
Z_lm_L_Doxo_EG

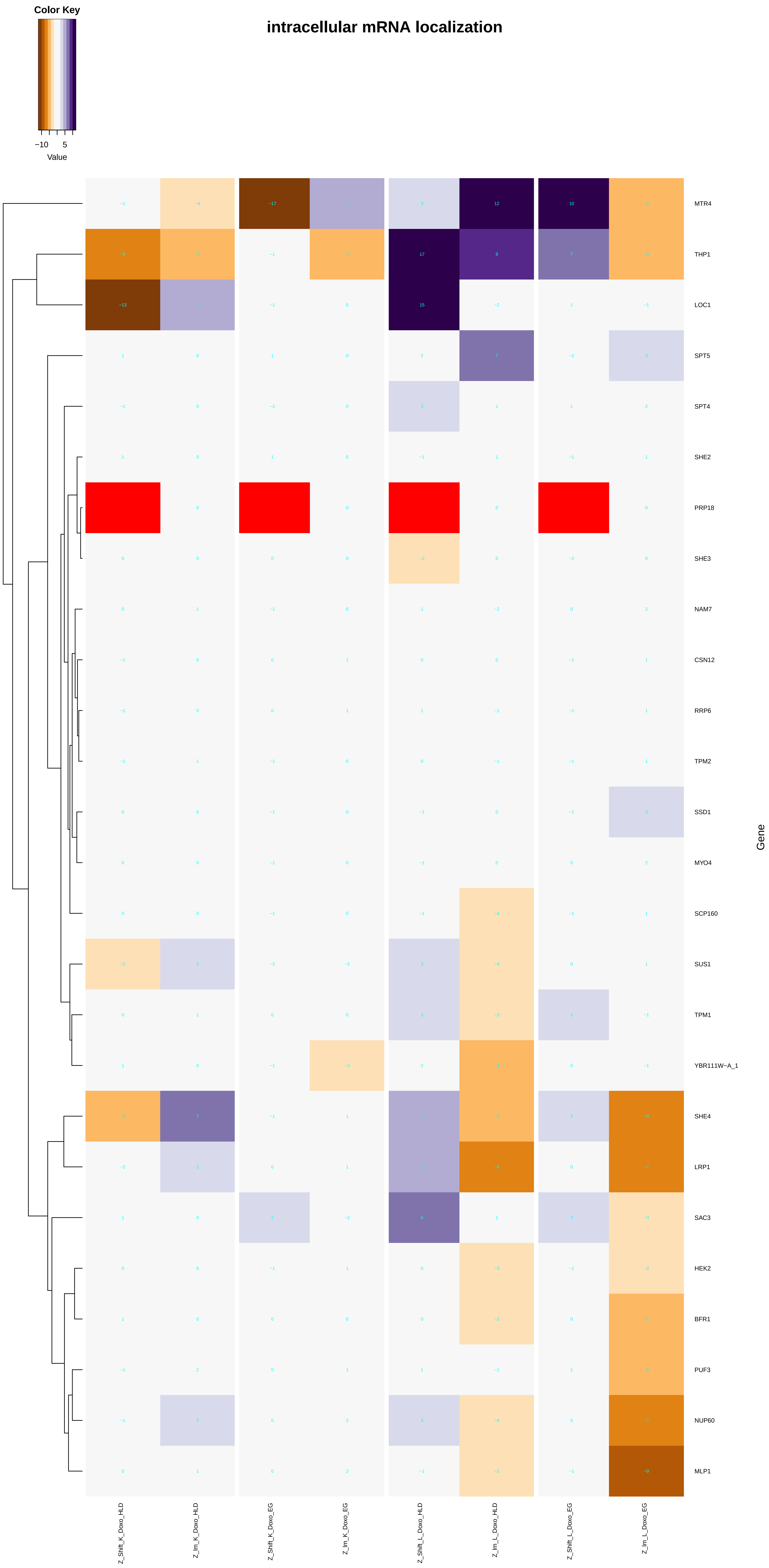
intracellular mRNA localization
Color Key
−10
5
Value
MTR4
THP1
LOC1
SPT5
SPT4
SHE2
PRP18
SHE3
NAM7
CSN12
RRP6
TPM2
SSD1
MYO4
SCP160
SUS1
TPM1
YBR111W−A_1
SHE4
LRP1
SAC3
HEK2
BFR1
PUF3
NUP60
MLP1
Gene
Z_Shift_K_Doxo_HLD
Z_Im_K_Doxo_HLD
Z_Shift_K_Doxo_EG
Z_Im_K_Doxo_EG
Z_Shift_L_Doxo_HLD
Z_Im_L_Doxo_HLD
Z_Shift_L_Doxo_EG
Z_Im_L_Doxo_EG

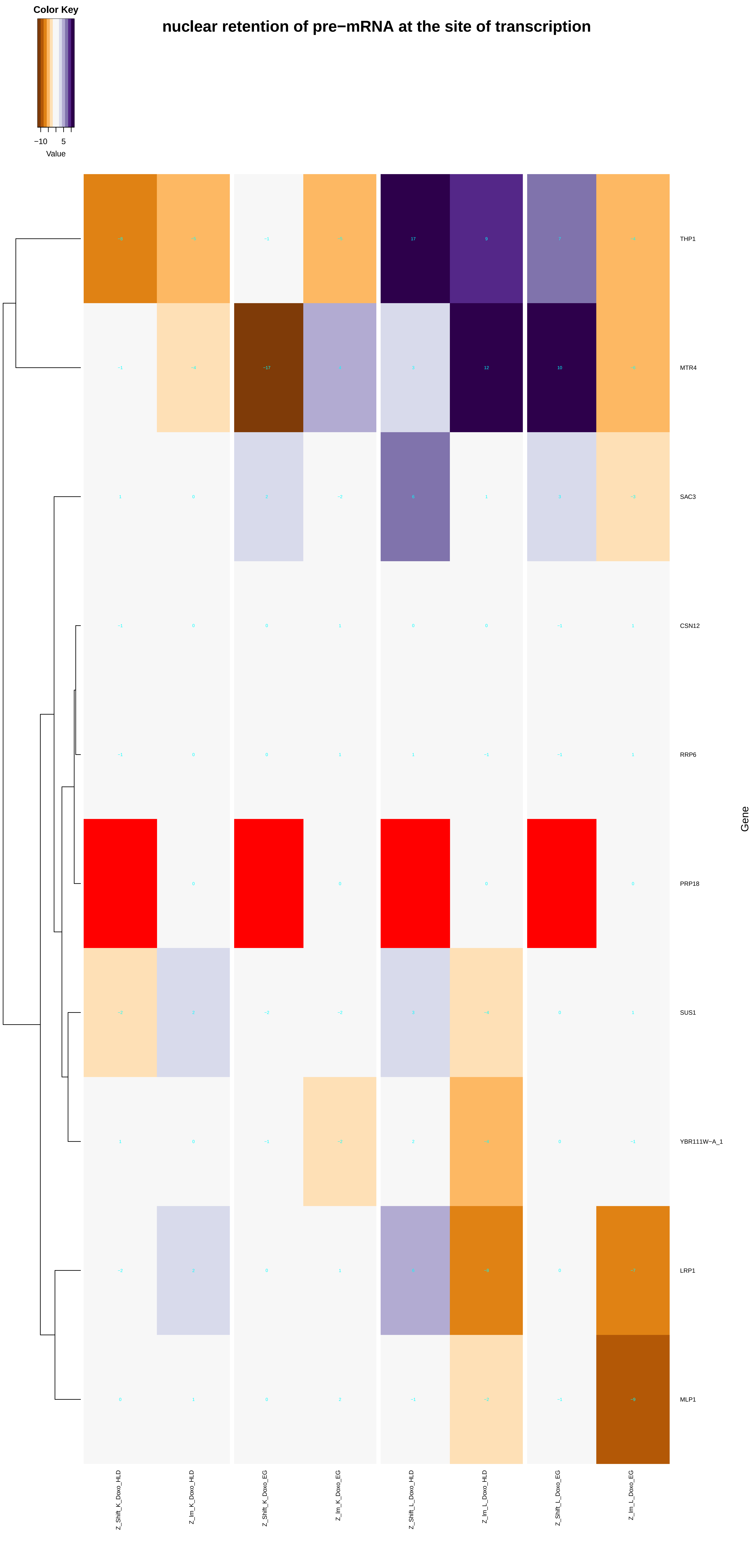
Color Key
−10
5
Value
nuclear retention of pre−mRNA at the site of transcription
THP1
MTR4
SAC3
CSN12
RRP6
PRP18
SUS1
YBR111W−A_1
LRP1
MLP1
Gene
Z_Shift_K_Doxo_HLD
Z_Im_K_Doxo_HLD
Z_Shift_K_Doxo_EG
Z_Im_K_Doxo_EG
Z_Shift_L_Doxo_HLD
Z_Im_L_Doxo_HLD
Z_Shift_L_Doxo_EG
Z_Im_L_Doxo_EG
